# 

**Published:** 1903-11-21

**Authors:** 


CONTENTS.

"The Terrible Imposture and Force of Words" 125

The Education of the Consumptive 126

Annotations 127

Notes and News 128

Hospital Clinics and Medical Progress—

Infective Endocarditis 129

Adherent Pericardium 129

Dilatation of the Bronchial Tubes in Children 130

Non-medicinal Treatment of Constipation 130

The Physical Factors in Photo-therapy 131

Progress in Disease of Digestive Organs 132

Progress in Neurology 133

Progress in State Medicine 134

New Appliances and Things Medical 135

The Book World of Medicine and Science 136

Hospital Administration—

British Institutions for the Care of the Inebriate—IV. 137

Danish Medical Institutions 138

The Future of St. Bartholomew's Hospital 139

Hospital Meetings 140

Editor's Letter-Box 141

Scraps and Gleanings 142

The Student of Medicine 142

*NURSING SECTION.*

Notes on News from the Nursing World—

The King of Italy and the Italian Hospital—Lord Roberts and his Nurses—Bristol General Hospital and the Pension Fund—The Reply of the Authorities of Dudley Hospital—A Warning to Nursing Associations—National Memorial to Nursing Sisters—Nursing at Ladywell Workhouse—Girls from School as Probationers—Tragic Death of a Nurse—Nurses and Total Abstinence—Compliment to a Courageous Nurse—Resignation of a Matron—Nurse and Nursemaids—Presentation by Nurses to a Lady Mayoress—A Prosperous Scottish Association—Short Items 99-102

Lectures on Ophthalmic Nursing 103

The Nurses of Bristol General Hospital 104

Wants and Workers 106

Everybody's Opinion 106

Travel Notes and Queries 107

Appointments 107

Presentations 107

Novelties for Nurses 107

Echoes from the Outside World 108

A Book and its Story 109

For Reading to the Sick 110

Notes and Queries 110

